# The correlation between speckle-tracking echocardiography and coronary angiography in suspected coronary artery disease with normal left ventricular function

**DOI:** 10.34172/jcvtr.2022.30520

**Published:** 2022-12-17

**Authors:** Krishan Yadav, Jayesh Prajapati, Gaurav Singh, Iva Patel, Ajay Karre, Pradeep Kumar Bansal, Vicky Garhwal

**Affiliations:** ^1^Yatharth Super Speciality Hospital, Noida, UP, India; ^2^Department of Cardiology, U. N. Mehta Institute of Cardiology and Research Centre (UNMICRC), Civil Hospital Campus, Asarwa, Ahmedabad-380016, Gujarat, India; ^3^Department of Research U. N. Mehta Institute of Cardiology and Research Centre (UNMICRC), Civil Hospital Campus, Asarwa, Ahmedabad-380016, Gujarat, India

**Keywords:** Speckle Tracking Echocardiography, Global Longitudinal Peak Systolic Strain, Suspected Stable Angina Pectoris

## Abstract

***Introduction:*** Our study objects to determine the diagnostic accuracy of two-dimensional speckle tracking echocardiography (2DSTE) in predicting presence and severity of coronary artery disease (CAD).

***Methods:*** Patients with stable angina pectoris with normal left ventricular function (>50%) undergoing coronary angiography were enrolled and subjected to speckle tracking echocardiography. Global longitudinal peak systolic strain was measured and correlated to the results of coronary angiography for each patient.

***Results:*** Number of male (*P*=0.001), diabetes (*P*=0.01) and smoking (*P*=0.01) patients were significantly higher in the CAD group compared to non-CAD patients. Global longitudinal peak systolic strain (GLPSS) was significantly (*P*=0.0001) lower in CAD patients in comparison to non- CAD patients. GLPSS showed significantly lower in patients with Syntax score (SS)≥22 in comparison to SS<22. Cut-off value -19 for GLPSS could be used to predict the presence of significant CAD with 80.6% sensitivity and 76.5% specificity (area under curve (AUC) -0.83, *P*=0.0001). The mean GLPSS value decreased as the number of diseased coronary vessels increased (*P*=0.0001). The optimal cut-off value of -16 GLPSS with a sensitivity of 76.7% and specificity of 83.3% [AUC 0.84, *P*<0.0001] was found significant to predict CAD severity. Multivariate regression of GLPSS and another risk factor for predicting significant CAD, GLPSS showed OR=1.55 (CI-1.36-1.76) *P*=0.0001 for predicting the presence of CAD.

***Conclusion:*** 2DSTE can be used as a non-invasive screening test in predicting presence, extent and severity of significant CAD patients with suspected stable angina pectoris.

## Introduction

 Coronary artery disease (CAD) is a major cardiac concern leading to morbidity and mortality worldwide. Previously published studies have reported death rate of that 40% in the urban and 30% in rural areas of India is due to cardiovascular diseases (CVD).^1^ Hence, it is very important to identify the coronary artery disease before development of significant coronary stenosis, which leads to major morbidity and mortality. However, for patient with normal systolic function, usually the threshold for investigation, especially invasive tests are higher. The early detection and treatment are required to prevent major adverse cardiac complications. The non-invasive technique with ability to predict the presence of significant coronary stenosis can help to diagnose the presence of CAD, leads to a decrease in associated significant events.

 For patients with suspected cardiac diseases the dominant non-invasive cardiac imaging techniques are ECG, Echocardiography, tread mill test (TMT), computerized tomography (CT) coronary, TMT and nuclear stress test. Although diagnosing by conventional echocardiography adds a little information in identifying patients with suspected stable angina due to normal motion at rest, except there is a history of prior myocardial infarction or myocardial stunning. CT coronary angiography exposes patients to the risk of radiation and contrast agents. The treadmill test is also not always possible in the old age group or those with baseline ST-segment and T-wave changes in ECG. The nuclear stress test is costly and not widely available.^2,3^

 Speckle Tracking Echocardiography (STE) when imaged by ultrasound is one of the technique that recognizes the motion of cardiac tissues in the heart using speckle pattern in the cardiac muscles. The myocardial fibres with maximum chances of ischemia are the longitudinally orientated fibres that are located subendocardial, where intermittent ischemia leads to myocardial stunning can be detected by strain measurement using speckle tracking.^4-6^ Measurements of longitudinal motion and deformation are consequently, the most sensitive markers compared to the other parameter for coronary artery disease. Left ventricular longitudinal strain measurement by two-dimensional (2D) speckle-tracking echocardiography (2D STE) has been shown a suitable technique for predicting significant CAD and acute or subacute ischemia.^7-10^

 The present study aimed to assess and predict the value of global longitudinal peak systolic strain (GLPSS) performed at rest and its relation with presence, extent and severity of CAD in patients with suspected stable angina pectoris with normal left ventricular function in the Western Indian population.

## Materials and methods

###  Study design and study population

**Figure 1:**
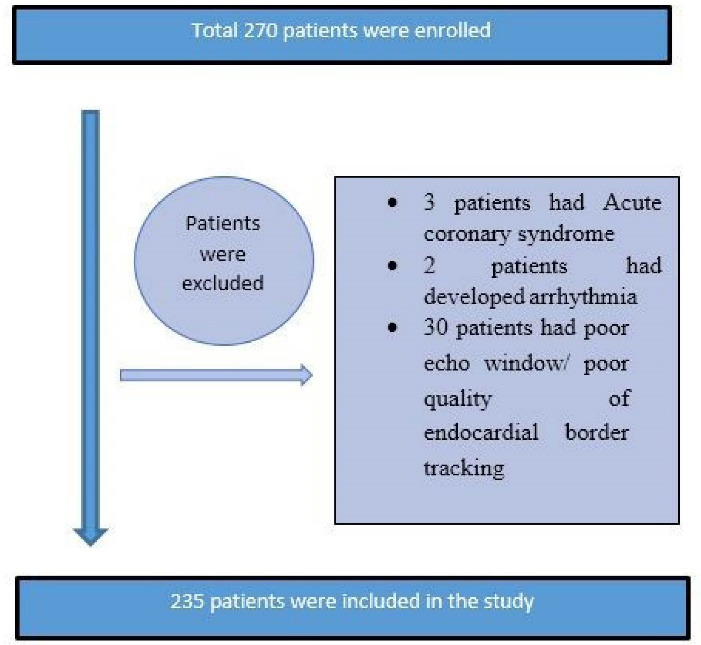
Study population flow chart.

 This prospective observational study was carried out in our tertiary cardiac care center from December 2018 to December 2020. The present study enrolled patients with stable coronary angina with normal left ventricular function ( > 50%). The institutional ethics committee approved the study (IEC number. UNMICRC/CARDIO/2018/06). The informed consent was taken from all the participants. Patients were selected for study who fulfilled all inclusion & exclusion criteria. The purpose of the study was explained to the patient and informed consent was obtained. The flow chart of the study is shown in Figure 1.

 Inclusion criteria: Patients (≥ 18 years) admitted with complaints and history suggestive of suspected stable angina with normal left ventricular function ( ≥ 50%).

 Exclusion criteria: Patients with acute coronary syndrome (ACS), previous history of presence of CAD, congestive heart failure & left ventricular (LV) systolic dysfunction (ejection fraction (EF) < 50%), atrial fibrillation or premature ventricular contraction, any valvular disease, left bundle-branch block, poor acoustic window, patients on cytotoxic drug therapy and patients who refused to sign informed consent were excluded from the study.

 All patients underwent routine clinical examination, which included detailed signs and symptoms at presentation, medical history, physical examination, and assessment of CVD status, and the results were recorded in a pre-designed data collection form. The patients participating in the study were contacted only by the persons involved in the study. All the patient-specific data were kept in strict confidence.

###  Transthoracic Echocardiographic evaluation

 Transthoracic echocardiography was performed with commercially available systems iE 33 xMatrix (Philips Healthcare, Andover, MA, USA) and images were taken through a 3.5 MHz transducer and stored. Two experienced cardiologist has performed the measurements in all study patients. LV diameters were measured from the parasternal long-axis view. We used Simpson’s biplane method to calculate the LV end-diastolic and end-systolic volumes using two- and four-chamber apical views and LVEF was subsequently measured.

###  Speckle-tracking strain analysis

 Speckle-tracking analysis was used to calculate LV GLS. The speckle tracking technique measures the quantification of LV myocardial deformation by tracking on standard greyscale two-dimensional (2D) images, frame-to-frame, natural acoustic markers that interference patterns from sub-wavelength structures throughout the myocardium. Longitudinal strain, evaluating the shortening (negative strain) and lengthening (positive strain) of the myocardial wall, was measured from the three apical views, i.e. 4-chamber, 3- chamber and 2-chamber views then stored loops were selected independently and available software was applied for strain measurement. LV endocardial border was manually traced in the end-systolic frame followed by automatically calculated longitudinal strain value which is global strain and not the average value of each segment strain and respective curves were obtained. The most susceptible area of myocardium to ischemia is subendocardium which is longitudinally orientated fibers, so we measured longitudinal deformation, which is the most sensitive markers of CAD ^11^. During the entire cardiac cycle the strain curve which is obtained peak negative value was considered as peak strain value.

###  Statistical analysis 

 All study analysis were performed using SPSS vs. 20 (Chicago,IL,USA). Quantitative and qualitative variables were expressed as the mean ± standard deviation and number (%) respectively. A comparison of values between two groups was performed using the independent sample *t*-test for continuous variables and chi-square test used for categorical variables. Logistic regression was used to predict the different risk factors for the presence of coronary artery disease. The predictive diagnostic value of GLPSS for presence and severity of coronary artery disease was calculated using receiver operating characteristic (ROC) curve. A nominal significance was taken as a two-tailed *P *value < 0.05.

## Results

 From the total number of 235 patients, 102 (43.40%) patients had normal coronary artery and 133 (56.59%) had coronary artery disease. The number of Patients with the single-vessel disease were 64 (48.12%), the Double vessel disease were 31 (23.31%), the Triple vessel disease was 38 (28.57%).

Table 1 Baseline characteristic of population: Table. 1 represents the demographic characteristic of the population. Male to female ratio was 3.8:1 in CAD patients. Age (*P* = 0.03), BMI (*P* = 0.01), Diabetes (*P* = 0.02) and smoking (*P* = 0.01) were significantly higher in the CAD group. Other baseline variables were found evenly distributed between two groups.

**Table 1:** Baseline variables

**Variables**	**CAD absent (N=102)**	**CAD Present (N=133)**	***P*** ** value**
Male	58 (36.3%)	102 (63.8%)	0.001
Female	44 (58.7%)	31 (41.3%)	0.14
Age group	52.70 ± 11.21	55.86 ± 10.78	0.03
BMI	24.74 ± 3.31	26.34 ± 5.86	0.01
Hypertension	29 (28.4%)	45 (33.8%)	0.38
Diabetes	11 (10.8%)	32 (24.1%)	0.01
Dyslipidemia	1 (1%)	3 (2.3%)	0.45
Smoking	6 (5.9%)	21 (15.8%)	0.02
Family history	4 (3.9%)	2 (1.5%)	0.24
ECG (ST-T changes)	8 (7.8%)	14 (10.5%)	0.48
Ejection fraction	56.41 (2.27%)	56.02 (2.748%)	0.25

Abbreviations: CAD, coronary artery disease; BMI, body mass index.

Table 2 represents the mean global longitudinal peak systolic strain (GLPSS) in both groups. The mean global longitudinal peak systolic strain (GLPSS) value was significantly (*P* = 0.0001) decreased as the number of diseased vessels were increased in CAD patients in comparison to non-CAD patients. GLPSS value was significantly (*P* = 0.0001) decreased in patients with syntax score ≥ 22 (GLPSS = -14.72. ± 2.05).

**Table 2:** Mean GLPSS value in patients with no. of vessels blocked

**Mean GLPSS value**			
	**CAD absent **	**CAD present **	***P*** ** value **
GLPSS mean	-20.29 ± 2.47	-16.66 ± 2.81	0.0001
**No. of vessels blocked**			
Single vessel disease GLPSS	-20.29 ± 2.47	-17.45 ± 2.68	0.0001
Double vessel disease GLPSS	-20.29 ± 2.47	-16.56 ± 2.22	0.0001
Multivessel disease GLPSS	-20.29 ± 2.47	-14.65 ± 2.12	0.0001

Abbreviations: CAD, coronary artery disease; GLPSS, Global longitudinal peak systolic strain.

**Figure 2:**
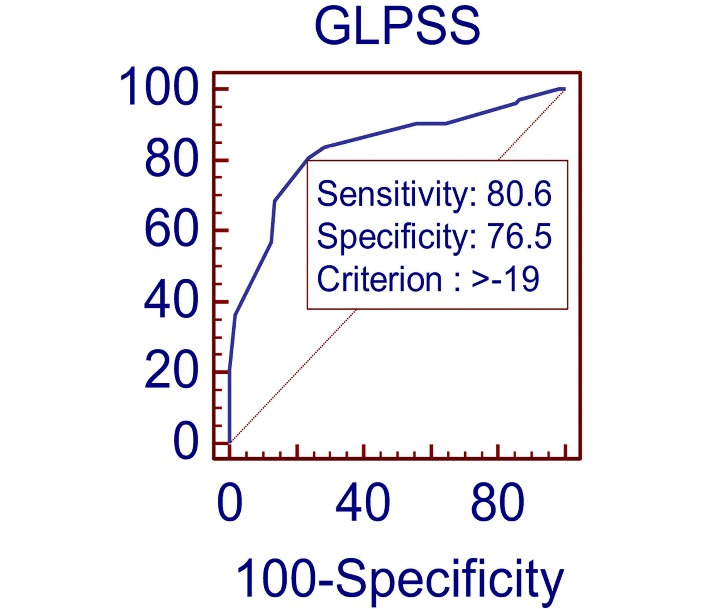
ROC curve of GLPSS value to predict the significant CAD.

 GLPSS cut-off value > -19 can be used to predict the significant CAD with sensitivity of 80.5 and specificity of 76.5%. (AUC 0.83, 95% CI 0.77–0.88, *P* = 0.0001) as shown in Figure 2.

Table 3 presents the GLPSS cutoff value for number of diseased vessels and severity of CAD. -20 value of GLPSS predicted the single vessel disease (SVD) with 79.69% sensitivity and 70.27% specificity (area under curve (AUC) = 0.78, *P* < 0.0001). GLPSS cutoff value of -18, -16 and -16 with 77.78% and 86.49%, 81.82% and 98.20%, 76.7% and 83.33% with (AUC = 0.87, *P* < 0.0001), (AUC = 0.94, *P* < 0.0001), (AUC = 0.84, *P* < 0.0001) predicated DVD, TVD and Syntax score ≥ 22 respectively.

**Table 3:** Different GLPSS cut-off for predicting significant and severity of CAD

	**GLPSS** **cut-off value**	**Sensitivity**	**Specificity**	**AUC (95% CI)**	***P*** ** value**
CAD	-19	80.6%	76.5%	0.83(0.77-0.88)	< 0.0001
SVD	-20	79.69%	70.27%	0.78(0.71-0.86)	< 0.0001
DVD	-18	77.78%	86.49%	0.87(0.81-0.92)	< 0.0001
TVD	-16	81.82%	98.20%	0.94(0.89-0.97)	< 0.0001
SYNTAX ≥ 22	-16	76.7%	83.33%	0.84(0.76-0.90)	< 0.0001

Abbreviations: SVD, single vessel disease; DVD, double vessel disease; TVD, triple vessel disease; CAD, coronary artery disease; CI, confidence interval.

**Figure 3:**
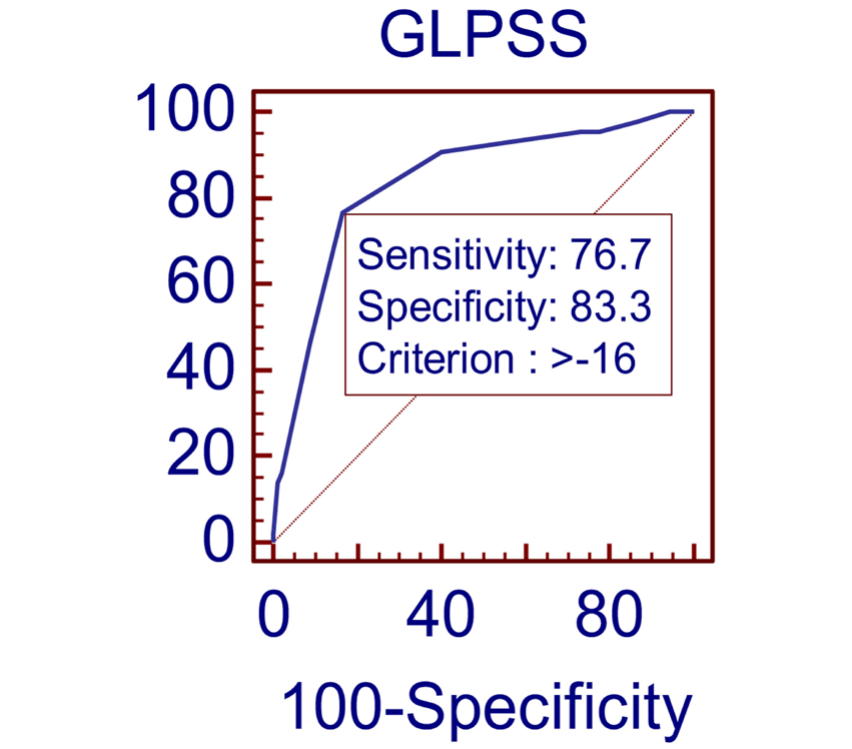
ROC curve of GLPSS value for CAD severity.

Figure 3 represents the ROC curve of GLPSS value for CAD severity. GLPSS value of -16 predicted CAD severity with sensitivity of 76.7% and specificity of 83.3% (AUC 0.84, 95% CI 0.76–0.90 *P* < 0.0001)

 The logistic regression analysis showed that GLPSS and different risk factors for the predictor of presence of CAD (Table 4). GLPSS with the odds ratio of 1.367 (95% CI -1.37-1.76) and *P *value < 0.0001 was an independent significant predictor of the presence of significant CAD.

**Table 4:** Regression analysis for prediction of CAD

**Variables in the Equation**					
	**B**	***P*** ** value**	**Odd- ratio**	**95% CI**	
				**Lower**	**Upper**
Age	0.018	0.228	1.018	0.989	1.048
BMI	0.032	0.499	1.033	0.941	1.134
Diabetes	0.144	0.754	1.155	0.468	2.848
Smoking	0.659	0.242	1.933	0.640	5.836
GLPSS	0.440	0.0001	1.552	1.367	1.762

Abbreviations: CI, confidence interval; BMI, body mass index; GLPSS, global longitudinal peak systolic strain.

**Figure 4:**
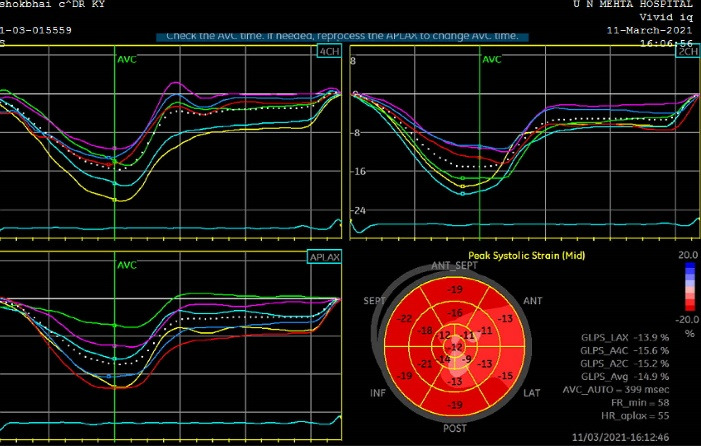
Echocardiographic image of GLPSS.

**Figure 5:**
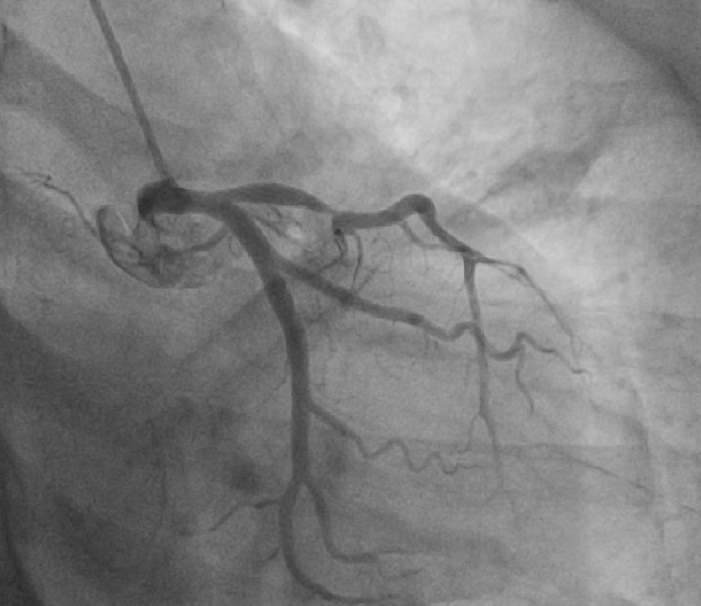
Coronary angiography image (same patient image with echocardiography image shown).

Figure 4 and Figure 5 show the echocardiographic image of GLPSS and Coronary angiography of same patient respectively.

## Discussion

 In this study, we investigated the global longitudinal peak systolic strain (GLPSS) value through speckle tracking echocardiography (STE) to predict the presence and severity of coronary artery disease in stable coronary angina patients. There was a negative correlation between the GLPSS value and the severity of coronary artery disease.

 Of the total 235 patients in the present study, 102 (43.4%) patients had normal coronary angiography and 133 (56.6%) had significant CAD compared to the other study the ratio between normal coronary and significant CAD was 1/3rd in our study. This might be due to the questionnaire followed at our institute had more sensitivity, than specificity which gives us a strong control group. The present study showed the mean age group of 59.49 ± 11.1 years, which was similar to the mean age population 60 ± 12 reported by Montgomery et al^12^ We found a significantly higher number of patients with advanced age, male gender, BMI, diabetes and smoking in the CAD group in comparison to the non-CAD group which is consistent with previously reported studies.^12-14^

 In the present study, we found mean GLPSS value of -17.45 ± 2.64 in patients with SVD, -16.56 ± 2.22 in patients with double vessel disease (DVD) and -14.65 ± 2.12 for multivessel disease patient, while in a study by Gaibazzi et al^15^ found -22 ± 1.5 (SVD), -19.4 ± 2.4 (DVD) and -18 ± 2.3(TVD) in CAD patients and Radwan et al^16^ reported GLPSS value of -15.13 ± 0.64 (SVD), -12.25 ± 0.9 (DVD) and -9.1 ± 1.94 (TVD), which supports our study trend of inverse correlation between GLPSS value and severity of coronary artery disease.

 In the current study, GLPSS value showed sensitivity for detection of the number of diseased vessels with cut-off value -20 for single-vessel CAD (79.69% sensitivity and 70.27% specificity, AUC: 0.783); -18 for two vessels disease (77.78%, sensitivity 86% specificity, AUC: 0.87) and -16 for three vessels CAD (81.82% sensitivity and specificity 98.20% AUC 0.94) which supports the result reported by Biering-Sørensen et al^11^, that showed GLPSS value gradually declined with increasing severity of CAD defined by increasing number of stenotic coronary vessels.

 We found a significant (*P* < 0.0001) difference in GLPSS among Non-CAD (-20.29 ± 2.47) and CAD (-16.66 ± 2.8), which are very consistent with the results of Montgomery et al ^12^ and Bakhoum et al.^17^

 Moustafa et al^13^ found cutoff value of GLPSS for SVD, DVD, TVD, high syntax score (> 16) -18.44 (sensitivity 90%,specificity 95.1%); -17.35(sensitivity 90%,specificity 88.9%%); -15.33(sensitivity 63%,specificity 72%); -13.75(sensitivity 80%,specificity 91%) respectively which supports the present study cutoff value for SVD is > -20 with sensitivity of 79.69 % and 70.27% specificity, DVD (-18%) with sensitivity of 77.70% and specificity of 86.49%, TVD (> -16%) with sensitivity of 81.82% and 98.20% specificity and high syntax (> 22) > -16 with sensitivity 76.7% and specificity 83.33. This might be because of inter vendor and inter observer variability.

 Abdelrazek et al^14^ found GLPSS cut-off value for high syntax score (≥ 22) was -16.5 (sensitivity 93%, specificity 91%), which is similar to the present study GLPSS cut-off -16 with sensitivity of 81.82% and 98.20% specificity for syntax score ≥ 22.

 The SYNTAX score is used to grade lesion complexity for coronary revascularization. Most studies have reported longitudinal strain correlates with the presence and severity of CAD, but limited data has shown the correlation between GLPSS and Syntax score.

 Study by Tanaka et al ^18^ reported a moderate correlation between SYNTAX scores and the extent of stress-induced myocardial ischemia as measured on myocardial SPECT (r = 0.647, *P* < 0.0001) in patients without prior myocardial infarction. These significant correlations were predominantly based on patients with a low SYNTAX score (r = 0.580, *P* < 0.0001), whereas such a correlation found insignificant for patients with an intermediate-high SYNTAX score (r = –0.033). Dogdus M et al^19^ defined severe coronary artery disease by Gensini score ≥ 20 and he reported the cut-off value of GLS for severe CAD was -10 (sensitivity 88.9%, specificity 92.9). In our study, we found inverse correlation between GLPSS and syntax score (r = 0.534, *P* < 0.000), indicating more severe the CAD more severely affected GLPSS.

 It is undeniable that the number of patients enrolled in our study was relatively small and not randomized. For the comparison of value of GLPSS with presence and severity of disease we used coronary angiography technique only. One type of the echocardiography equipment (2D strain software iE 33 matrix) was used. GLPSS Strain which is influenced by left ventricular mass, hemodynamic variables, and software type, in addition to myocardial ischemia. All these factors could be potential confounders. Radial, transverse, circumferential strain and synchrony analysis were not achieved in the present study.

## Conclusion

 Our study recommends that addition of GLPSS value using speckle tracking echocardiography to the standard echocardiography protocol for assessment of myocardial ischemia in patients with suspected coronary artery disease can predict patients likely to have more severe and significant CAD, which avoids significant invasive coronary angiography and revascularization, still the values of GLPSS should be tested and analyzed in larger population studies.

## Acknowledgments

 We thank all our patients and clinical staff of our institute for supporting us to complete the work.

## Author Contributions

**Conceptualization: **Jayesh Prajapati, Krishan Yadav.

**Methodology:** Jayesh Prajapati, Krishan Yadav, Gaurav Singh.

**Validation: **Jayesh Prajapati, Krishan Yadav, Gaurav Singh.

**Formal Analysis:** Krishan Yadav, Iva Patel.

**Investigation: **Jayesh Prajapati, Krishan Yadav, Gaurav Singh.

**Resources: **U.N.Mehta Institute of Cardiology and Research Centre.

**Data Curation:** Iva Patel, Krishan Yadav.

**Writing—Original ft Preparation:** Iva Patel.

**Writing—Review and Editing:** Krishan Yadav, Iva Patel, Jayesh Prajapati.

**Visualization:** Iva Patel, Ajay Karee, Pradeep Kumar Bansal, Vicky Garhwal.

**Project Administration:** Jayesh Prajapati, Krishan Yadav, Iva Patel.

**Funding Acquisition:** U.N.Mehta Institute of Cardiology and Research Centre.

## Funding

 U. N. Mehta Institute of Cardiology and Research Centre (UNMICRC), Civil Hospital Campus, Asarwa, Ahmedabad-380016, Gujarat, India.

## Ethical Approval

 The institutional ethics committee approved the study (IEC number. UNMICRC/CARDIO/2018/06).

## Competing Interests

 All authors have none to declare.
